# Glycomic and sialoproteomic data of gastric carcinoma cells overexpressing ST3GAL4

**DOI:** 10.1016/j.dib.2016.03.022

**Published:** 2016-03-14

**Authors:** Stefan Mereiter, Ana Magalhães, Barbara Adamczyk, Chunsheng Jin, Andreia Almeida, Lylia Drici, Maria Ibáñez-Vea, Martin R. Larsen, Daniel Kolarich, Niclas G. Karlsson, Celso A. Reis

**Affiliations:** a I3S - Instituto de Investigação e Inovação em Saúde, University of Porto, Portugal; b Institute of Molecular Pathology and Immunology of the University of Porto - IPATIMUP, Porto, Portugal; c Institute of Biomedical Sciences of Abel Salazar - ICBAS, University of Porto, Portugal; d Department of Medical Biochemistry and Cell Biology, Institute of Biomedicine, Sahlgrenska Academy, University of Gothenburg, Sweden; e Department of Biomolecular Systems, Max Planck Institute of Colloids and Interfaces, 14424 Potsdam, Germany; f Free University Berlin, Berlin, Germany; g Department of Biochemistry and Molecular Biology, University of Southern Denmark, Odense, Denmark; h Medical Faculty, University of Porto, Portugal

**Keywords:** N-glycome, O-glycome, Gastric cancer, Sialyltransferase, Sialoproteome

## Abstract

Gastric carcinoma MKN45 cells stably transfected with the full-length *ST3GAL4* gene were characterised by glycomic and sialoproteomic analysis. Complementary strategies were applied to assess the glycomic alterations induced by ST3GAL4 overexpression. The *N*- and *O*-glycome data were generated in two parallel structural analyzes, based on PGC-ESI-MS/MS. Data on glycan structure identification and relative abundance in ST3GAL4 overexpressing cells and respective mock control are presented. The sialoproteomic analysis based on titanium-dioxide enrichment of sialopeptides with subsequent LC-MS/MS identification was performed. This analysis identified 47 proteins with significantly increased sialylation. The data in this article is associated with the research article published in Biochim Biophys Acta “Glycomic analysis of gastric carcinoma cells discloses glycans as modulators of RON receptor tyrosine kinase activation in cancer” [1].

**Specifications Table**

**Table:** 

Subject area	*Biology*
More specific subject area	*Glycobiology in cancer*
Type of data	*Tables*
How data was acquired	*N-and O-glycome analysed by PGC-LC ESI MS/MS performed on an LTQ ion trap mass spectrometer and PGC-nanoLC ESI MS/MS performed on an amaZon ETD Speed ion trap. Sialoproteome performed using Easy-nLC II system and Orbitrap Fusion Tribrid system.*
Data format	*Analyzed*
Experimental factors	*MKN45 cells stably transfected with the full-length ST3GAL4 gene or empty vector (mock).*
Experimental features	*MKN45 cells grown in vitro in RPMI medium with 10% FBS.*
Data source location	*Not applicable*
Data accessibility	*Data is within this article.*

**Value of the data**

• Data shows the *N*- and *O*-glycome of the MKN45 gastric carcinoma cells and ST3GAL4 sialyltransferase overexpressing cells.
• Data provides a list of glycoproteins with altered sialylated *N*-glycans in ST3GAL4 overexpressing cells, including several cancer associated proteins.
• These data are valuable as a source for novel biomarkers for gastric cancer.

## 1 Data

This data article includes the *N*- and *O*-glycome of MKN45 cells and assesses the glycosylation alterations induced by ST3GAL4 overexpression. In addition, we provide the data on the proteins with significant increased sialylated *N*-glycans upon ST3GAL4 overexpression.

## 2 Experimental design, materials and methods

Glycomic and sialoproteomic analyses were performed as described below comparing MKN45 cells stably transfected with the full-length *ST3GAL4* gene or empty vector (mock). Glycome data and statistical evaluation are shown in Table 1, Table 2, Table 3, Table 4. Sialoproteome data and statistical evaluation are shown in Table 5, Table 6.

## 3 *N*-glycomic strategy I: sample preparation and PGC LC-ESI-MS/MS

Frozen cell pellets (10^7^ cells) of mock or *ST3GAL4* transfected MKN45 cells [2] were directly resuspended in 7 M urea, 2 M thiourea, 40 mM Tris, 2% CHAPS, 10 mM DTT and 1% protease inhibitor (Sigma-Aldrich, St. Louis, MO). The cell membranes were disrupted by 10 times 10 s sonication with 16 amplitudes and 1 min on ice in between, and subsequent shaking at 4 °C overnight. To reduce the viscosity of the lysates, the DNA was degraded by adding 1 µl benzonase® nuclease (250 units, Sigma-Aldrich) and 30 min incubation at 37 °C. In order to impair refolding of proteins, 25 mM iodoacetamide were added for alkylation during 1 h in the dark. The lysates were centrifuged for 30 min at 14,000 rpm and the supernatants were transferred to a fresh tube.

Then, solubilized proteins were concentrated by adding 150 µl of supernatant on a 10 kDa cut-off spinfilter (PALL, Port Washington, NY), spinning down for 5 min with 12,000xg and washing 3 times with 100 µl 50 mM NH_4_HCO_3_, pH 8.4. *N*-linked oligosaccharides were released in the spinfilter using 20 µl 50 mM NH_4_HCO_3_ and PNGase F (5 mU, Prozyme, Hayward, CA) with incubation at 37 °C overnight. Subsequently, the *N*-glycans were collected by washing 3 times with 20 µl H_2_O and dried in Speedvac. Reactions were quenched with 1 µl of glacial acetic acid and *N*-glycan samples were desalted and dried as previously described [3]. *N*-glycan samples were subjected to LC-ESI-MS/MS analysis using a 10 cmx250 µm I.D. column, prepared in-house, containing 5 µm porous graphitized carbon (PGC) particles (Thermo Scientific, Waltham, MA). Glycans were eluted using a linear gradient from 0% to 40% acetonitrile in 10 mM NH_4_HCO_3_ over 40 min at a flow rate of 10 μl/min. The eluted *N*-glycans were detected using a LTQ ion trap mass spectrometer (Thermo Scientific) in negative-ion mode with an electrospray voltage of 3.5 kV, capillary voltage of −33.0 V and capillary temperature of 300 °C. Air was used as a sheath gas and mass ranges were defined dependent on the specific structure to be analyzed. The data were processed using the Xcalibur software (version 2.0.7, Thermo Scientific) and manually interpreted from their MS/MS spectra.

Optionally prior to analysis *N*-glycan were digested in 50 mM sodium phosphate, pH 6.0 at 37 °C overnight using sialidase S (4 mU, ProZyme) that releases α2-3 linked non-reducing terminal sialic acids (recombinant sialidase from *Streptococcus pneumoniae*, expressed in *Escherichia coli*) and sialidase A (5 mU, ProZyme) that releases α2-3/6/8 linked non-reducing terminal sialic acid (recombinant gene from *Arthrobacter ureafaciens*, expressed in *Escherichia coli*) to confirm sialic acid linkage.

All analyzes were performed in three independent replicates and results were subjected to statistical analyses (Average, standard deviation and unpaired *t*-test) Table 1.

## 4 *N*-glycomic strategy II: sample preparation and PGC nanoLC-ESI MS/MS

Frozen cell pellets (10^7^ cells) of mock or *ST3GAL4* transfected MKN45 cells were directly resuspended in 2 mL of lysis buffer (50 mM Tris–HCl, 100 mM NaCl, 1 mM EDTA and protease inhibitor at pH 7.4) and stored on ice for 20 min. The cells were lysed using a Polytron homogenizer for at least three times for 10 s in a cold room. Cellular debris and unlysed cells were sedimented by centrifugation at 2000*g* for 20 min at 4 °C. The supernatant was collected and the pellets resuspended in 1 mL of lysis buffer and centrifuged again at 2000*g* for 20 min at 4 °C. All the supernatants were combined, diluted in 20 mM Tris–HCl (pH 7.4) and ultracentrifuged at 120,000*g* for 90 min at 4 °C. The supernatant was separated from the pellet containing the cell membrane proteins. The membrane proteins were resuspended with 150 μL of 100 mM ammonium bicarbonate buffer and lyophilized overnight. The dried samples were solubilized in 50 μL of 8 M urea and 10 µL aliquots were dot-blotted onto PVDF membranes as described previously [4]. *N*- and *O*-glycan release as well as PGC-nanoLC ESI MS/MS analysis on an amaZon ETD Speed ion trap (Bruker, Bremen, Germany) were performed as described in detail previously [4], [5] (Table 2).

## 5 *O*-glycomic strategy I: sample preparation and LC-ESI-MS/MS

After the removal of *N*-glycan, as previously described in “1. *N*-glycomic strategy I: Sample preparation and LC-ESI-MS/MS”, the *O*-linked glycans were released from retained glycoproteins in spinfilter using reductive β-elimination (0.5 M NaBH_4_, 50 mM NaOH at 50 °C, 16 h). Reactions were quenched with 1 µl of glacial acetic acid and glycan samples were desalted and dried as previously described [3]. Glycans were subjected to LC-ESI-MS/MS analysis using a 10 cmx250 µm I.D. column, prepared in-house, containing 5 µm porous graphitized carbon (PGC) particles (Thermo Scientific. Waltham. MA). Glycans were eluted using a linear gradient from 0% to 40% acetonitrile in 10 mM NH_4_HCO_3_ over 40 min at a flow rate of 10 μl/min. The eluted *O*-glycans were detected using a LTQ ion trap mass spectrometer (Thermo Scientific) in negative-ion mode with an electrospray voltage of 3.5 kV, capillary voltage of −33.0 V and capillary temperature of 300 °C. Air was used as a sheath gas and mass ranges were defined dependent on the specific structure to be analyzed. The data were processed using the Xcalibur software (version 2.0.7. Thermo Scientific) and manually interpreted from their MS/MS spectra (Table 3).

## 6 *O*-glycomic strategy II: sample preparation and PGC nanoLC-ESI MS/MS

Sample preparation and analysis were performed as previously described in Section 2. “*N*-glycomic strategy II: Sample preparation and PGC nanoLC-ESI MS/MS” (Table 4).

## 7 Sialoproteomic analysis

### 7.1 Cell lysis, protein digestion and iTRAQ labeling

Cell pellets were redissolved in ice-cold Na_2_CO_3_ buffer (0.1 M, pH 11) supplemented with protease inhibitor (Roche complete EDTA free), PhosSTOP phosphatase inhibitor cocktail (Roche) and 10 mM sodium pervanadate on ice. The suspensions were tip probe sonicated for 20 s (amplitude=50%) twice and incubated at 4 °C for 1 h. The lysates were then centrifuged at 100,000×*g* for 90 min at 4 °C to enrich membrane proteins (pellet). The pellets were washed with 50 mM triethylammonium bicarbonate (TEAB) to remove any remaining soluble protein. Membrane fraction was resuspended directly in 6 M urea and 2 M thiourea, reduced in 10 mM DTT for 30 min and then alkylated in 20 mM IAA for 30 min at room temperature in the dark.

Samples were incubated with endoproteinase Lys-C (Wako, Osaka, Japan) for 2 h (1:100 w/w). Following the incubation, the samples were diluted 8 times with 50 mM TEAB (pH 8) and trypsin was added at a ratio of 1:50 (w/w) and left overnight at room temperature. Trypsin digestion was stopped by the addition of 2% formic acid and then the samples were centrifuged at 14,000×*g* for 10 min to precipitate any lipids present in the sample. The supernatant was purified using in-house packed staged tips with a mixture of Poros R2 and Oligo R3 reversed phase resins (Applied Biosystem, Foster City, CA, USA). Briefly, a small plug of C18 material (3 M Empore) was inserted in the end of a P200 tips, followed by packing of the stage tip with the resins (resuspended in 100% ACN) by applying gentle air pressure. The acidified samples were loaded onto the micro-column after equilibration of the column with 0.1% trifluoroacetic acid (TFA), washed twice with 0.1% TFA and peptides were eluted with 60% ACN/0.1% TFA. A small amount of purified peptides (1 μl) from each sample was subjected to Qubit assay to determine the concentration, while the remaining samples were dried by vacuum centrifugation. Afterwards, peptides were redissolved in dissolution buffer and a total of 150 μg for each condition was labeled with 4-plex iTRAQ^TM^ (Applied Biosystems, Foster City, CA) as described by the manufacturer. After labeling, the samples were mixed 1:1:1:1 and lyophilized by vacuum centrifugation.

### 7.2 Sialic acid containing glycopeptide enrichment by TiSH protocol

The method used for sialylated glycopeptides enrichment is a modification of the TiSH protocol [6] described in [7], [8]. Briefly, samples were resuspended in loading buffer (1 M glycolic acid, 80% ACN, 5% TFA) and incubated with TiO_2_ beads (GL Sciences, Japan, 10 μm; using a total of 0.6 mg TiO_2_ beads per 100 μg of peptides). The supernatant containing the un-modified peptides was carefully separated. The TiO_2_ beads were sequentially washed with loading buffer, washing buffer 1 (80% ACN, 1% TFA) and washing buffer 2 (20% ACN, 0.1% TFA), saving the washings with the previous supernatant. The bound peptides were eluted with 1.5% ammonium hydroxide by shaking for 15 min. The eluted fraction containing the phosphopeptides and sialylated glycopeptides was dried by vacuum centrifugation and subjected to an enzymatic deglycosylation in 20 mM TEAB buffer using 500 U of PNGase F (New England Biolabs, Ipswich, MA) and 0.1 U of Sialidase A (Prozyme, Hayward, CA) overnight at 37 °C.

To separate phosphorylated peptides and formerly glycosylated peptides, the samples were subjected to a second TiO_2_ enrichment procedure to separate phosphorylated from deglycosylated peptides. The supernatant containing the deglycosylated peptides was saved and the beads were washed with 50% ACN, 0.1% TFA. The washing was added to the supernatant. The deglycosylated fraction was desalted on Oligo R3 staged tip column and dried prior to the HILIC fractionation [7]. All fractions were dried by vacuum centrifugation prior nLC-MS/MS analysis.

### 7.3 Sialic acid containing glycopeptide analysis by nLC-MS/MS

Samples were resuspended in 6 µL of 0.1% TFA for analysis. Peptides were loaded on an in-house packed Reprosil-Pur C18-AQ (2 cmx100 µm, 5 µm; Dr. Maisch GmbH, Germany) pre-column and separated on an in-house packed Reprosil-Pur C18-AQ (17 cmx75 µm, 3 µm; Dr. Maisch GmbH, Germany) column using an Easy-nLC II system (Thermo Scientific, Bremen, Germany) and eluted at a flow of 250 nL/min. Mobile phase was 95% acetonitrile (B) and water (A) both containing 0.1% formic acid. Depending on the samples, gradient was from 1% to 30% solvent B in 80 or 110 min, 30–50% B in 10 min, 50–100% B in 5 min and 8 min at 100% B. Mass spectrometric analyses were performed in an Orbitrap Fusion Tribrid system (Thermo Scientific, Bremen, Germany). MS scans (400–1200 m/z) were acquired in the orbitrap at a resolution of 120000 at 200 m/z for a AGC target of 5×10^5^ ions and a maximum injection time of 60 ms. Data-dependent HCD MS/MS analysis at top speed of the most intense ions were performed at a resolution of 30000 at 200 m/z for a AGC target of 5×10^4^ and a maximum injection time of 150 ms using the quadrupole to isolate the ions and an isolation window of 1.2 m/z, a NCE of 38% and a dynamic exclusion of 20 s.

The raw data were processed and quantified by Proteome Discoverer (version 1.4.1.14, Thermo Scientific) against SwissProt and Uniprot human reference databases by using Mascot (v2.3.02, Matrix Science Ltd, London, UK) and Sequest HT, respectively. Database searches were performed using the following parameters: precursor mass tolerance of 10 ppm, product ion mass tolerance of 0.02 Da, 1 missed cleavages for trypsin, carbamidomethylation of Cys and iTRAQ labeling on protein *N*-terminal and Lys as fixed modifications, and phosphorylation on S/T/Y and deamidation of Asn as dynamic modifications. The iTRAQ datasets were quantified using the centroid peak intensity with the “reporter ions quantifier” node. Only peptides with up to a *q*-value of 0.01 (Percolator), Mascot and Sequest HT rank 1, Sequest HT ΔCn of 0.1, cut-off value of Mascot score ≥ 18 and a cut-off value of XCorr score for charge states of +1, +2, +3, and +4 higher than 1.5, 2, 2.25 and 2.5, respectively, were considered for further analysis.

### 7.4 Data normalization and significance analysis

Three biological replicates were analyzed and submitted to the statistical analysis. The log2 values of the measured intensities were normalized by the median. Modified peptides were merged with the R Rollup function (http://www.omics.pnl.gov) allowing for one-hit-wonders and using the mean of the normalized intensities for each peptide. Quantification of proteins was obtained by merging the un-modified peptides with the R Rollup function considering at least 2 unique peptides not allowing for one-hit-wonders and using the mean of the intensities. Then the mean over the experimental conditions for each peptide in each replicate was subtracted in order to merge data from different iTRAQ runs. Formerly sialylated glycopeptides containing the consensus motif for *N*-linked glycosylation (NXS/T/C; where X # P) were normalized based on the protein expression in each of the replicates. Significant up/down-regulations between experimental conditions were calculated allowing a false discovery rate of 0.05. Therefore, we applied combined limma and rank product tests [9], subsequently corrected for multiple testing according to Storey.

Since spontaneous deamidation is frequently observed for asparagine residues, especially when the C-terminal amino acid is glycine (NG), the sites with NGS/T/C are considered as only potential glycosylation. However, in order to reduce the contribution from spontaneous deamidation in the final list, we sort first for the *N*-linked consensus site (NXS/T/C) and then we filter for proteins that are membrane-associated in order to exclude intracellular proteins that are not *N*-linked glycosylated (Table 5, Table 6).

## Figures and Tables

**Table 1:** Identified *N*-glycan structures I.






Structures are represented by the mass to charge ratio (*m*/*z*) in which they were identified and quantified, by monosaccharide composition and proposed structure based on MS/MS analyses. The relative quantities were determined by base-peak intensity of extracted ion chromatograms. The average value (Avg) and standard deviation (SD) of triplicates are shown, as well as the *p*-value (*t*-test; *p<0.05; **p<0.01; ***p<0.001). Increased or decreased relative abundance are shown in red or blue, respectively. Structures marked as not analyzed (na) were either not detected or overlapped with other structures in given sample, precluding their quantification. Unknown linkage is represented by “?”.

**Table 2:** Identified *N*-glycan structures II.







Structures are represented by their [M–H] value and charge state in which they were identified and quantified, by monosaccharide composition and proposed structure based on MS/MS analyzes. The relative quantities were determined by base-peak intensity of extracted ion chromatograms. The ratio of glycan abundance in ST3GAL4 transfected cells relative to mock transfected is shown on the right column. Increases or decreases larger than 2 fold in glycan abundance are highlighted red or blue, respectively.

**Table 3:** Identified *O*-glycan structures I.




Structures are represented by the mass to charge ratio (*m*/*z*) in which they were identified and quantified, by monosaccharide composition and proposed structure based on MS/MS analyzes. The relative quantities were determined by base-peak intensity of extracted ion chromatograms. The average value (Avg) and standard deviation (SD) of triplicates are shown, as well as the *p*-value (*t*-test; *p<0.05; **p<0.01). Increased or decreased relative abundance are shown in red or blue, respectively. Unknown linkage is represented by “?”.

**Table 4:** Identified *O*-glycan structures II.




Structures are represented by their [M–H] value and charge state in which they were identified and quantified, by monosaccharide composition, type of core and proposed structure based on MS/MS analyzes. The relative quantities were determined by base-peak intensity of extracted ion chromatograms. The ratio of glycan abundance in *ST3GAL4* transfected cells relative to mock transfected is shown on the right column. Increases or decreases greater than 1.5 fold in glycan abundance are highlighted red or blue, respectively.

**Table 5:** Proteins with increased *N*-glycan sialylation.

Accession number	Protein name	Sequence	*N*-glycan site	ST3GAL4 /Mock	Limma&Rank (*p*-value)	Func. Grp.
O15031	Plexin-B2	ISVAGRNdeCSFQPER	N844	2.89	0.0342	RS
ALSNdeISLR	N127	2.08	0.0358
O60637	Tetraspanin-3	TYNdeGTNdePDAASR	N127	2.96	0.0358	RS
TYNdeGTNPDAASR	N127	2.98	0.0433
O75882	Attractin	GICNdeSSDVR	N300	2.35	0.0342	IR
P02786	Transferrin receptor protein 1	KDFEDLYTPVNdeGSIVIVR	N251	1.72	0.0433	TT
P05026	Sodium/potassium-transporting ATPase subunit beta-1	FKLEWLGNdeCSGLNDETYGYK	N158	2.79	0.0479	TT
P06213	Insulin receptor	HNdeLTITQGK	N445	2.45	0.0479	RS
P06731	Carcinoembryonic antigen-related cell adhesion molecule 5	TLTLFNdeVTR	N204	2.67	0.0342	AM
TLTLFNVTRNdeDTASYK	N208	3.11	0.0342
P06756	Integrin alpha-V	ISSLQTTEKNdeDTVAGQGER	N874	4.02	0.0342	AM
NdeMTISR	N554	4.02	0.0342
P07602	Prosaposin	TNdeSTFVQALVEHVKEECDR	N215	2.59	0.0479	O
P08962	CD63 antigen	CCGAANdeYTDWEK	N150	1.94	0.0394	RS
P10909	Clusterin	EIRHNdeSTGCLR	N291	2.12	0.0342	O
P11117	Lysosomal acid phosphatase	QTPEYQNdeESSR	N177	3.05	0.0383	E
P12821	Angiotensin-converting enzyme	IGLLDRVTNdeDTESDINYLLK	N445	2.62	0.0456	P
P13473	Lysosome-associated membrane glycoprotein 2	LNdeSSTIK	N275	3.09	0.0342	AM
VASVINdeINPNdeTTHSTGSCR	N253	1.96	0.0358
VASVININPNdeTTHSTGSCR	N257	2.06	0.0456
P13688	Carcinoembryonic antigen-related cell adhesion molecule 1	NdeQSLPSSER	N363	2.76	0.0479	AM
P13726	Tissue factor	RNdeNTFLSLR	N169	5.52	0.0342	P
NdeNTFLSLR	N169	5.76	0.0358
P18564	Integrin beta-6	EVEVNdeSSK	N463	3.71	0.0479	AM
P21589	5׳-nucleotidase	LDNdeYSTQELGK	N333	1.67	0.0479	E
P26006	Integrin alpha-3	ELAVPDGYTNdeRTGAVYLCPLTAHK	N86	2.68	0.0433	AM
P30825	High affinity cationic amino acid transporter 1	LCLNNdeDTK	N235	3.48	0.0297	TT
LCLNdeNdeDTK	N234	3.01	0.0342
P35613	Basigin	ALMNdeGSESR	N268	2.91	0.0342	P
P42892	Endothelin-converting enzyme 1	NdeSSVEAFK	N632	1.89	0.0394	P
DYYLNdeKTENEK	N270	5.64	0.0479
P43007	Neutral amino acid transporter A	VVTQNdeSSSGNdeVTHEK	N201	2.58	0.0477	TT
P46059	Solute carrier family 15 member 1	NdeDSCPEVK	N562	4.66	0.0358	TT
P48960	CD97 antigen	WCPQNSSCVNdeATACR	N38	1.87	0.0479	AM
P54760	Ephrin type-B receptor 4	CAQLTVNdeLTRFPETVPR	N203	2.34	0.0342	RS
Q04912	Macrophage-stimulating protein receptor	LPEYVVRDPQGWVAGNdeLSAR	N841	3.26	0.0358	RS
Q08380	Galectin-3-binding protein	DAGVVCTNdeETR	N125	2.43	0.0358	AM
Q08722	Leukocyte surface antigen CD47	SDAVSHTGNdeYTCEVTELTREGETIIELK	N111	6.51	0.0056	AM
Q11206	ST3GAL4	LFGNdeYSR	N61	5.77	0.0088	O
Q12913	Receptor-type tyrosine-protein phosphatase eta	HGSNdeHTSTYDK	N582	3.15	0.0342	RS
VSDNdeESSSNdeYTYK	N391	2.94	0.0342
VSDNESSSNdeYTYK	N396	2.74	0.0383
GPNdeGTEGASR	N525	2.76	0.0427
IHVAGETDSSNdeLNdeVSEPR	N411	1.69	0.0479
SNdeDTAASEYK	N142	2.77	0.0479
Q13641	Trophoblast glycoprotein	CVNRNdeLTEVPTDLPAYVR	N81	1.85	0.0429	RS
Q14108	Lysosome membrane protein 2	ANIQFGDNdeGTTISAVSNdeK	N105	2.03	0.0394	T
ANdeIQFGDNdeGTTISAVSNK	N99	1.78	0.0479
Q15043	Zinc transporter ZIP14	ALLNHLDVGVGRGNdeVTQHVQGHR	N77	2.42	0.0479	TT
Q15758	Neutral amino acid transporter B(0)	NdeITGTR	N212	2.93	0.0479	TT
Q7Z7H5	Transmembrane emp24 domain-containing protein 4	QYGSEGRFTFTSHTPGDHQICLHSNdeSTR	N117	2.50	0.0479	T
Q8N271	Prominin-2	ILRNdeVSECFLAR	N725	3.11	0.0433	O
Q92542	Nicastrin	RPNdeQSQPLPPSSLQR	N417	1.66	0.0483	P
Q92673	Sortilin-related receptor	LTIVNdeSSVLDRPR	N871	1.92	0.0479	T
SRNdeSTVEYTLNK	N1986	1.67	0.0479
Q9BXB1	Leucine-rich repeat-containing G-protein coupled receptor 4	TLDLSYNNIRDLPSFNdeGCHALEEISLQR	N362	4.00	0.0358	RS
Q9BXS4	Transmembrane protein 59	LFSICQFVDDGIDLNdeRTK	N90	4.09	0.0342	T
Q9H330	Transmembrane protein 245	ILGDKVNdeNTAVIEK	N551	3.47	0.0433	O
Q9H5V8	CUB domain-containing protein 1	ASVSFLNFNdeLSNCERK	N270	3.33	0.0342	AM
IGTFCSNdeGTVSR	N180	2.93	0.0358
NdeVSGFSIANR	N205	2.33	0.0358
Q9HD43	Receptor-type tyrosine-protein phosphatase H	NdeATTAHNPVR	N203	6.86	0.0342	RS
ETRNdeATTAPNPVR	N381	2.93	0.0479
NdeTTNTSVTAER	N434	2.72	0.0479
Q9P2B2	Prostaglandin F2 receptor negative regulator	QRNdeNSWVK	N525	3.60	0.0429	RS
Q9UN76	Sodium- and chloride-dependent neutral and basic amino acid transporter B(0+)	SPIVTHCNdeVSTVNdeK	N174	2.44	0.0420	TT
Q9Y639	Neuroplastin	ANdeATIEVK	N229	2.59	0.0429	AM

List of significantly increased sialylated *N*-glycan modified peptides in the ST3GAL4 overexpressing cells compared to mock control shown with accession number, protein name, peptide sequence and the identified *N*-glycan site. The fold in increase, p-value and the protein׳s functional group are also presented. AM: Adhesion and migration; E: Metabolic enzyme; RS: Receptor and signaling; T: Trafficking; TT: Transmembrane transporter; P: Protease; O: Others.

**Table 6:** Proteins with decreased *N*-glycan sialylation.

Accession number	Protein name	Sequence	N-glycan site	ST3GalIV/Mock	Limma & rank
(*p*-value)
O14672	Disintegrin and metalloproteinase domain-containing protein 10	NdeISQVLEK	N439	0.57	0.0149
P07602	Proactivator polypeptide	NdeSTKQEILAALEK	N426	0.57	0.0474

List of significantly decreased sialylated *N*-glycan modified peptides in the ST3GAL4 overexpressing cells compared to mock control shown with accession number, protein name, peptide sequence and the identified *N*-glycan site, fold in increase and the *p*-value.
